# Viral vector-mediated upregulation of serine racemase expression in medial prefrontal cortex improves learning and synaptic function in middle age rats

**DOI:** 10.18632/aging.204652

**Published:** 2023-04-12

**Authors:** Brittney Yegla, Asha Rani, Ashok Kumar

**Affiliations:** 1 Department of Neuroscience, McKnight Brain Institute, University of Florida, Gainesville, FL 32611, USA

**Keywords:** aging, medial prefrontal cortex, serine racemase, D-serine, NMDA receptor, cognitive flexibility

## Abstract

An age-associated decrease in N-methyl-D-aspartate receptor (NMDAR)-mediated synaptic function contributes to impaired synaptic plasticity and is associated with cognitive impairments. Levels of serine racemase (SR), an enzyme that synthesizes D-serine, an NMDAR co-agonist, decline with age. Thus, enhancing NMDAR function via increased SR expression in middle age, when subtle declines in cognition emerge, was predicted to enhance performance on a prefrontal cortex-mediated task sensitive to aging. Middle-aged (~12 mo) male Fischer-344 rats were injected bilaterally in the medial prefrontal cortex (mPFC) with viral vector (LV), SR (LV-SR) or control (LV-GFP). Rats were trained on the operant attentional set-shift task (AST) to examine cognitive flexibility and attentional function. LV-SR rats exhibited a faster rate of learning compared to controls during visual discrimination of the AST. Extradimensional set shifting and reversal were not impacted. Immunohistochemical analyses demonstrated that LV-SR significantly increased SR expression in the mPFC. Electrophysiological characterization of synaptic transmission in the mPFC slices obtained from LV-GFP and LV-SR animals indicated a significant increase in isolated NMDAR-mediated synaptic responses in LV-SR slices. Thus, results of the current study demonstrated that prefrontal SR upregulation in middle age rats can improve learning of task contingencies for visual discrimination and increase glutamatergic synaptic transmission, including NMDAR activity.

## INTRODUCTION

The function of N-methyl-D-aspartate receptors (NMDARs) has a profound influence on synaptic plasticity, cognition, psychiatric diseases, and connectivity of neural networks [1, 2]. Hippocampal and prefrontal cortex-dependent synaptic plasticity and cognitive capacities decline with advancing age [3–12]. More specifically, age-related changes in NMDARs, including subunit expression, corresponding neurotransmission, and oxidation-reduction status, are associated with age-related cognitive deficits. Age-related declines in NMDAR subunit expression, such as prefrontal GluN1 mRNA [13] and GluN2B protein in the frontal and hippocampal regions [14], impact pharmacokinetics of glutamatergic transmission [15–17]. Reductions in NMDAR-mediated transmission significantly contribute to these age-related synaptic and cognitive impairments [18–26]. Using GluN2B knockout mice, loss of GluN2B in cortical and CA1 pyramidal neurons impaired NMDAR-mediated neurotransmission, decreased spine density in the CA1 of the hippocampus, and resulted in cognitive deficits dependent upon hippocampal and prefrontal function [27]. These neural and cognitive impairments align with those observed in aging. Conversely, upregulation of the GluN2B subunit recovers synaptic plasticity and spatial memory in aged animals [28, 29]. In addition to subunit expression changes with aging, NMDAR function is modulated by oxidation-reduction status, and a higher level of oxidative stress is associated with advancing age, as well as cognitive impairment [21, 30–32].

D-serine, which is the primary co-agonist required for full activation of synaptic NMDARs, also decreases with aging [33–39]. D-serine is critical to synaptic plasticity and cognitive capacities [33, 36, 40–42]. D-serine levels are dependent upon serine racemase (SR), which is the enzyme that converts L-serine to D-serine [43–45]; correspondingly, levels of SR decline during aging [36, 37]. Deletion of SR affects hippocampal networks by altering the excitatory/inhibitory balance [46]. When age-related reductions in D-serine are supplemented with exogenous D-serine, synaptic transmission recovers in aged rodents in a dose-dependent manner, normalizing to young levels [12, 34, 36, 42].

Thus, upregulation of SR expression, which increases D-serine levels, may have therapeutic potential by upregulating NMDAR function. The beneficial effects of elevating D-serine in aging have been examined in relation to synaptic and cognitive capacities in the hippocampus. However, the prefrontal cortex (PFC), which is a brain region sensitive to disruptions in aging, has not been extensively evaluated. The PFC is involved in complex behaviors including executive function, which encompasses cognitive flexibility, inhibition, attention, and working memory [47, 48]. Executive dysfunction for working memory and cognitive flexibility also arises in middle age [49–51] and subtle attentional deficits associated with NMDAR impairments have been noted in middle age [52]. However, it is unknown if these deficits are related to NMDAR function, or more specifically D-serine availability.

We hypothesized that augmenting SR expression within mPFC glutamatergic neurons would improve attention and cognitive flexibility in middle-aged rats and facilitate synaptic responses in the mPFC. Thus, for this study, SR expression was upregulated in pyramidal neurons of the mPFC through lenti-viral technology to enhance NMDAR function and evaluate its impact on cognitive flexibility and NMDAR-mediated synaptic transmission in middle-age rats. The results demonstrate that viral vector-mediated upregulation of SR in the mPFC of middle-aged rats resulted in efficient contingency acquisition during visual discrimination, potentially through enhanced attentional function. Further, electrophysiological recordings demonstrated that up-regulation of SR expression significantly augmented NMDAR-mediated synaptic responses recorded from the mPFC.

## RESULTS

### LV-SR infusion increased prefrontal SR expression

**Figure 1:**
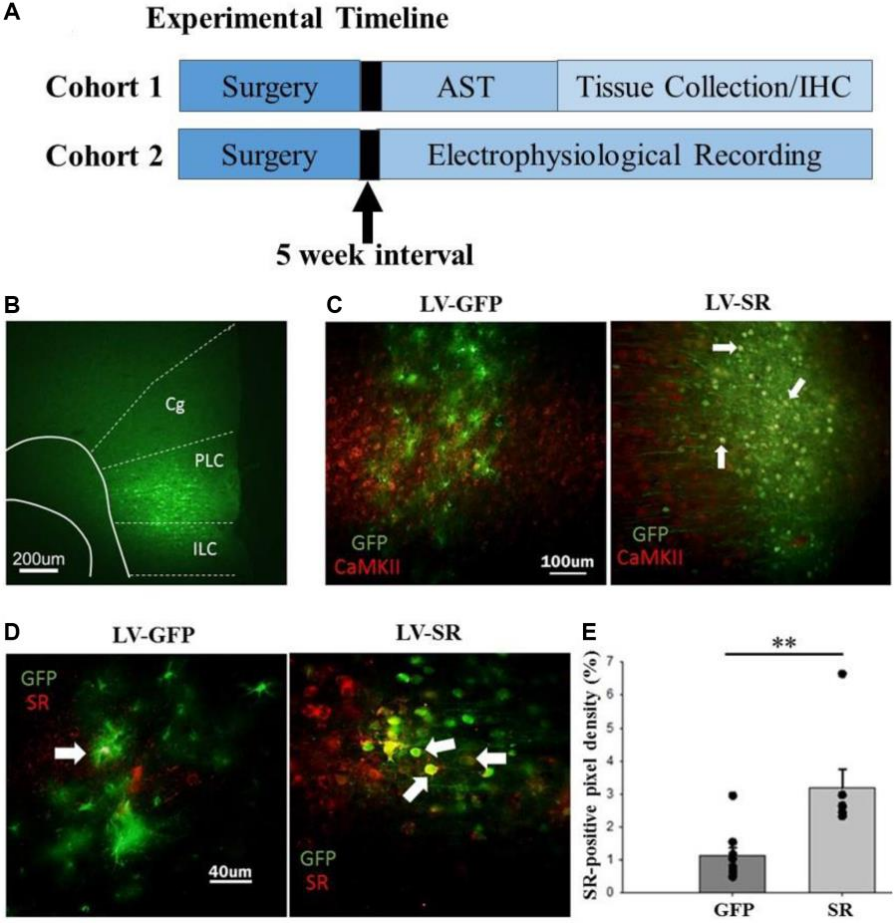
**Experimental timeline and confirmation of lentiviral transfection and upregulation of SR expression in mPFC.** (**A**) Experimental timeline illustrating details of experiments. Rats underwent surgery for lentiviral delivery into the mPFC and waited five weeks, to permit sufficient viral infection, before engaging in behavioral training on the attentional set shift task (AST) or electrophysiological recordings. Following AST, all rats were euthanized, had their tissue collected, and processed for immunohistochemistry (IHC). (**B**) Viral infection, represented as GFP+ expression, targeted the mPFC, which includes the cingulate (Cg), prelimbic (PLC), and infralimbic (ILC) cortices. (**C**) For rats injected with LV-SR (*N* = 7), viral infection (green, GFP) significantly overlapped with CaMKII+ cells (red; 52%). In contrast, rats injected with LV-GFP (*N* = 9), displayed limited overlap of GFP- (green) and CaMKII-positive cells (red). (**D**) Additional slices were evaluated for SR expression (red), and (**E**) based on further quantification (average of 4–7 slices/rat; individual rat’s average represented as single data point) showed that LV-SR significantly increased SR levels in the mPFC where the virus (GFP represented in green) was expressed. Data represented as mean ± SEM. ^**^p < 0.01.

To verify accurate expression of the SR viral vector, co-localization of GFP-positive cells and CaMKII-positive cells was evaluated within the mPFC (Figure 1B, 1C). Colocalization of GFP+/CaMKII+ cells was 52.48% in LV-SR-injected rats (*N* = 7). There was limited colocalization of GFP+/CaMKII+ cells in the control LV-GFP-injected rats (5.85%; *N* = 9). Injection of LV-SR increased the total area of fluorescence, indicative of SR levels, in the mPFC compared to LV-GFP-injected rats (*F*_(1,14)_ = 12.59, *p* < 0.01; Figure 1D, 1E).

### Up-regulation of SR expression accelerated visual discrimination learning

**Figure 2:**
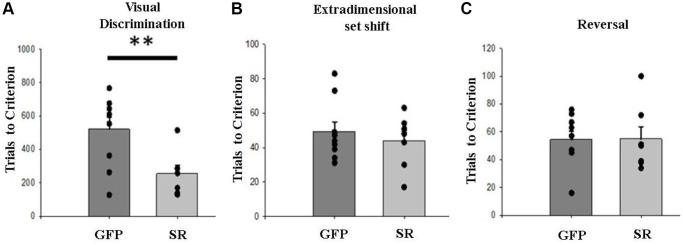
**Upregulation of SR expression in mPFC accelerated learning an initial rule.** (**A**) Middle-aged rats with LV-SR (*N* = 7) required fewer trials to reach criterion on visual discrimination than LV-GFP rats (*N* = 9). In contrast, LV-SR and LV-GFP rats did not significantly differ from one another regarding trials to criterion (TTC) for the extradimensional set shift (**B**) or reversal (**C**). Filled circles in (**A**–**C**) representing individual data points. Data represented as mean ± SEM, with individual rat’s performance displayed as single data point. ^**^p < 0.01.

Rats treated with LV-SR (*N* = 7) required fewer trials to attain criterion during the visual discrimination task (*F*_(1,14)_ = 8.23, *p* = 0.01; Figure 2A), suggesting that increased expression of SR accelerated learning of the task contingencies. LV-SR rats did not significantly differ from LV-GFP-treated rats (*N* = 9) for trials to criterion (TTC) when making an extradimensional shift during the set-shifting phase (*F*_(1,14)_ = 2.49, *p* = 0.14; Figure 2B), or a reversal (*F*_(1,14)_ = 0.25, *p* = 0.63; Figure 2C). Increasing SR levels also did not affect the number or the type of errors (i.e., perseverative, regressive, and never reinforced) made during set shift or reversal (*p* > 0.05). In addition, omissions did not vary by injection type (*p* > 0.05).

### SR upregulation augments basal synaptic transmission

**Figure 3:**
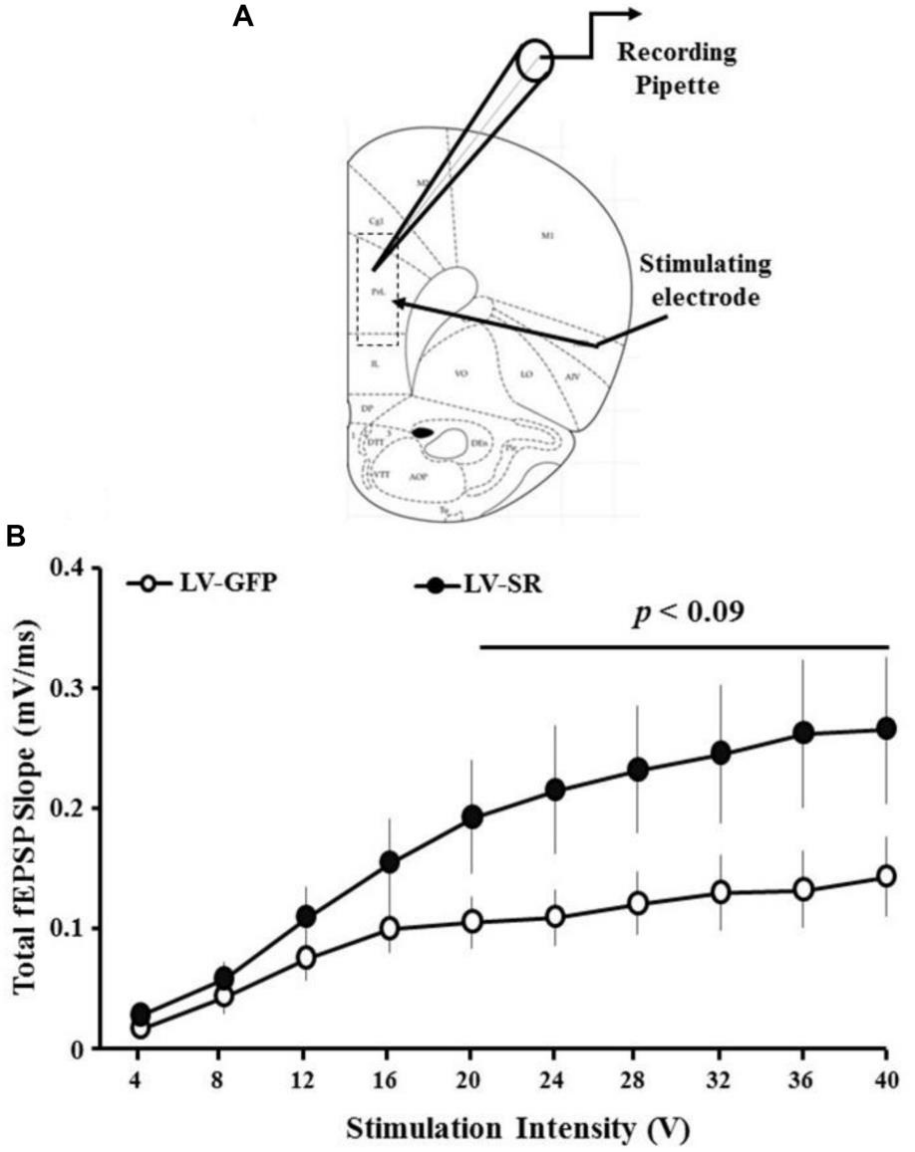
**Upregulation of SR increased total synaptic strength.** (**A**) Schematic of PFC slice adapted from Paxinos [108] illustrating location of recording and position of stimulating and recording electrodes. The rectangular dash lined box indicates the area used for recording total and NMDAR-mediated synaptic responses. (**B**) Input-output curve of total-fEPSP in slices obtained from LV-SR (*n* = 8/4 slices/animals) and LV-GFP (*n* = 8/4 slices/animals) animals, plotting the mean SEM slope across animals relative to increasing stimulation intensities. *p* < 0.09 represents a tendency for treatment effect at higher stimulation intensities (20–40V).

The effect of upregulation of SR expression on glutamatergic excitatory synaptic transmission was evaluated by recording and analyzing total fEPSPs from the mPFC slices in a subset of animals injected with LV-SR or LV-GFP vector. An input-output curve was generated by plotting the slope of the total synaptic response from LV-SR (*n* = 8/4 slices/animals) and LV-GFP (*n* = 8/4 slices/animals) rats as a function of increasing stimulation intensity (Figure 3). A repeated-measures ANOVA indicated main effects of increasing stimulation intensity (*F*_(9,126)_ = 23.43, *p* < 0.0001). A significant treatment by stimulation intensity interaction was observed (*F*_(9,126)_ = 3.04, *p* < 0.003) in absence of any main effect of treatment (Figure 3). However, repeated measures on higher stimulation intensities (20–40 volts) indicated that the total synaptic responses were higher in slices obtained from LV-SR-injected animals compared with LV-GFP, and there was a tendency for treatment effect (*F*_(1,14)_ = 3.28, *p* < 0.09).

**Figure 4:**
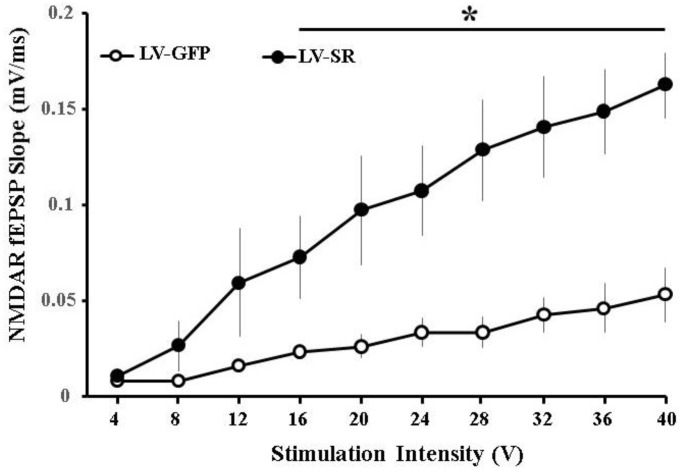
**Upregulation of SR increased NMDAR-mediated synaptic response.** Input-output curve of NMDAR-fEPSP in slices obtained from LV-SR (*n* = 8/4 slices/animals) and LV-GFP (*n* = 8/4 slices/animals) rats, plotting the mean SEM slope across animals relative to increasing stimulation intensities. ^*^indicates a significant treatment difference at higher stimulation intensities (16–40V).

The NMDAR-mediated synaptic component was pharmacologically isolated following the assessment of the total synaptic response. Input-output curves were constructed, and the synaptic response at each intensity was averaged across slices from the same animal (LV-SR: *n* = 8/4 slices/animals; LV-GFP: *n* = 8/4 slices/animals). An increase in the NMDAR-mediated synaptic response was observed for input-output curves plotting the NMDAR-fEPSPs slope as a factor of increasing stimulation intensity (Figure 4). A repeated-measures ANOVA indicated a significant main effect of stimulus intensity (*F*_(9,126)_ = 34.59, *p* < 0.0001) and treatment (*F*_(1,14)_ = 9.41, *p* < 0.0001) and an interaction of stimulation intensity and treatment (*F*_(9,126)_ = 10.32, *p* < 0.0001) on the slope of the recorded NMDAR-mediated synaptic responses. *Post hoc* analyses indicated that the NMDAR synaptic responses were significantly (*p* < 0.008) augmented in slices from LV- SR-injected animals when compared with LV-GFP (Figure 4).

### Redox regulation of NMDAR function

**Figure 5:**
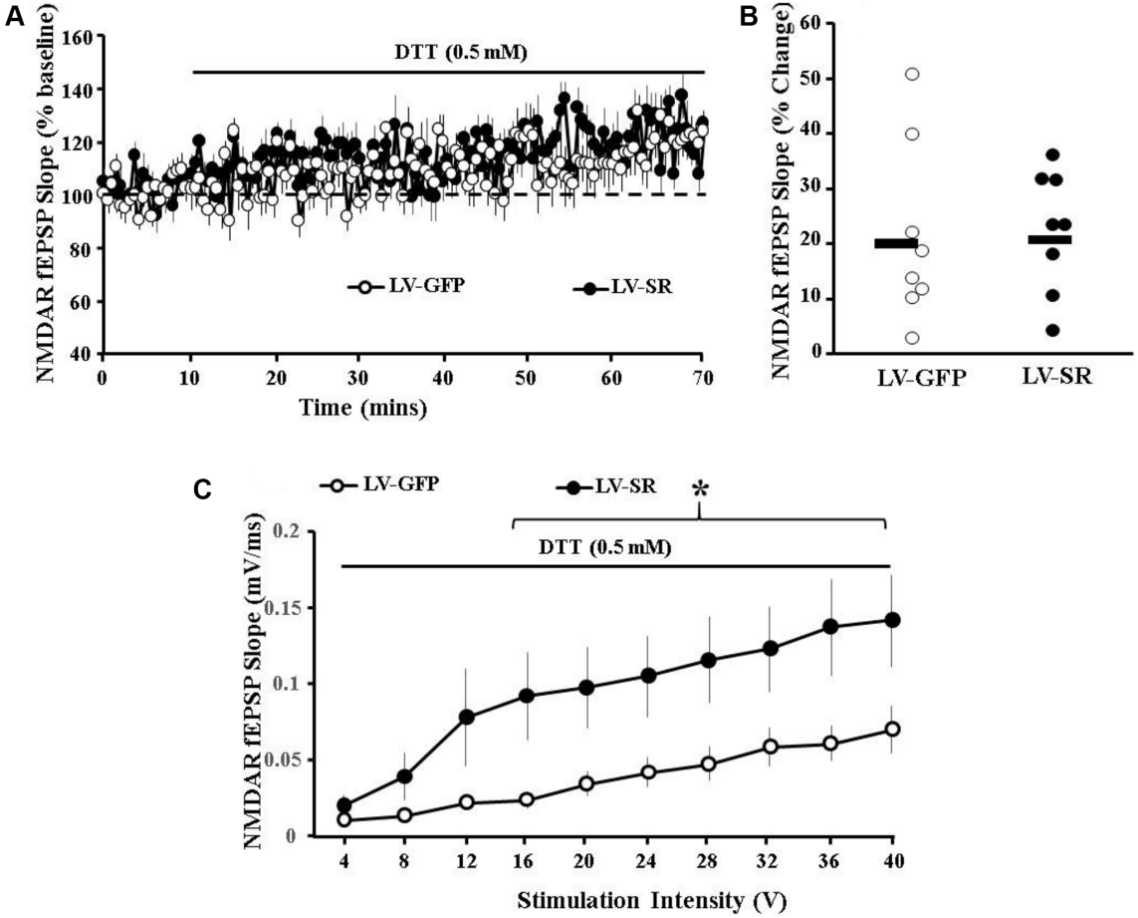
**Redox regulation does not contribute to increased NMDAR synaptic function induced by upregulation of SR expression.** (**A**) Time course of changes in the slope of the NMDAR-fEPSP obtained from mPFC slices 10 min before and up to 60 min after bath application of the reducing agent, DTT (0.5 mM; solid line) for slices obtained from LV-SR (filled circle, *n* = 8/4 slices/animals) and LV-GFP (control, open circle, *n* = 8/4 slices/animals) animals. (**B**) Scatter gram illustrating individual DTT-induced potentiation along with mean percentage increase (black solid line) in the slope of NMDAR-fEPSP in slices obtained from LV-SR (filled circle) and LV-GFP (open circle) animals. (**C**) Input-output curve of NMDAR-fEPSP slope in slices obtained from LV-GFP (open circle) and LV-SR (filled circle) rats 60 min following bath application of DTT (0.5 mm, solid line), across increasing stimulation intensities. ^*^indicates a significant treatment difference at higher stimulation intensities (16–40V).

Based on previous findings using middle-aged rats, redox state was identified as a potential modulator of NMDAR synaptic responses [21]. To determine whether the increased expression of SR interacted with redox state to alter the NMDAR synaptic response, NMDAR responses in the mPFC were isolated and the reducing agent DTT (0.5 mM) was applied to slices obtained from LV-SR and LV-GFP rats (Figure 5A). For LV-SR (*n* = 8/4 slices/animals) and LV-GFP (*n* = 8/4 slices/animals) animals, the NMDAR-fEPSP amplitude was maintained at ∼50% of maximum, and a stable baseline was recorded for at least 10 min. Subsequent application of DTT resulted in a significant increase in the synaptic response from the baseline for both LV-SR (*p* < 0.0007) and LV-GFP (*p* < 0.007) animals (Figure 5B). However, the bath application of DTT did not increase the NMDAR-fEPSP differentially from LV-SR rats (122.52 ± 3.89%) compared with LV-GFP animals (121.29 ± 5.76%), indicating involvement of non-redox regulation to account for the enhanced NMDAR synaptic function in the mPFC observed following upregulation of SR (Figure 5C).

## DISCUSSION

The results for the current study demonstrate that viral vector-mediated upregulation of SR, and putatively elevated D-serine levels in the mPFC, increased basal synaptic transmission in the PFC and improved acquisition of task contingencies for the visual discrimination condition. The lentiviral vector for SR, which was encoded with a CaMKII promoter, effectively infected glutamatergic pyramidal neurons in the PFC and increased SR expression at this site. Rats injected with LV-SR vector exhibited enhanced basal NMDAR-mediated synaptic transmission. These data suggest that the posited increase in D-serine from the elevated SR expression boosted NMDAR function in the mPFC of middle-aged rats.

In corroboration with these findings, D-serine has been identified as a critical co-agonist of NMDARs and is dependent on expression of SR, which converts L-serine to D-serine [43–45]. In the current study LV-SR, which colocalized with CaMKII-positive cells in the PFC, significantly increased SR expression and putatively increased D-serine levels. Surprisingly, LV-GFP did not exhibit strong colocalization with CaMKII-positive cells, despite also containing a CaMKII promoter, suggesting that the promoter was not selective in this specific viral vector, and is one weakness of this experiment. Both SR expression in neurons and L-serine shuttling in astrocytes are found to influence D-serine levels and alter CA1 synaptic neurotransmission [53]. D-serine may also be acting as an autocrine-signaling molecule, due to SR localization to postsynaptic regions of the neuron, including hippocampal pyramidal neurons and striatal GABAergic interneurons [54–57]. Moreover, despite the intention to target pyramidal neurons in the mPFC using the CaMKII promoter [58–60], this marker’s expression has recently been observed in parvalbumin or somatostatin interneurons from the superficial motor cortex of mice [61]. Thus, other neuronal types may have been transfected by LV-GFP or LV-SR and influenced SR expression. Thus, other neuronal types may have been transfected by LV-GFP or LV-SR and influenced SR expression. Notably, the LV-SR group exhibited significantly higher SR expression than the LV-GFP group, demonstrating that the LV-SR vector was efficacious.

In agreement with the hippocampal literature demonstrating the enhancing effects of D-serine on NMDAR-related function, the LV-SR group displayed increased basal NMDAR-mediated transmission in the mPFC. Previous studies have shown that application of D-serine enhanced evoked NMDAR-mediated currents in both hippocampal CA1 pyramidal neurons and interneurons, though to differing degrees potentially due to NMDAR subunit composition [62]. Endogenous sources of astrocytic D-serine also significantly contributed to NMDAR activity and long-term potentiation (LTP) [63]. Conversely, in SR knockout mice Ploux and colleagues (2020), amongst others [46, 64, 65], observed a weakening of NMDAR responses and LTP. Interestingly, this NMDAR hypofunction was observed in SR knockouts despite a natural compensatory mechanism of increased hippocampal expression of glycine, which also binds at the co-agonist site on the NMDAR.

Impaired NMDAR- and non-NMDAR-mediated glutamatergic transmission in the hippocampus during normal aging is strongly related to cognitive dysfunction, such as executive function and memory [66–68]. Engagement of the D-serine pathway has shown significant benefits in hippocampal-dependent NMDAR-mediated transmission and cognitive function in preclinical models of aging. In the aging brain, D-serine levels and NMDAR-mediated synaptic transmission are reduced [33–39], whereas supplementation with exogenous D-serine in aged rats [34] or mice with low D-serine release due to astrocytic mutations [69] normalize hypofunctional NMDAR responses. Moreover, the beneficial effects of sAPPα, a secreted form of amyloid precursor protein generated by the non-amyloidogenic pathway, on NMDAR-mediated transmission in hippocampal slices of aged mice are partially mediated by the D-serine pathway, as evidenced by an attenuation of these enhancing effects in SR knockout mice [70]. These beneficial effects of D-serine have translated to amelioration of cognitive function in elderly as well. Healthy older adults receiving D-serine supplementation exhibited improved spatial memory, learning, and problem solving, with greater effects observed for those with higher plasma D-serine levels [71]. Thus, D-serine bolsters glutamatergic transmission and cognitive function and could potentially attenuate age-related cognitive decline [72].

Initial cognitive impairments arising in aging include those dependent upon executive function, such as attention. To evaluate if increased expression of SR could improve one such cognitive capacity (i.e., cognitive flexibility) in middle-aged rats, we measured visual discrimination, attentional set shifting, and reversal learning. Set shifting is primarily dependent upon the mPFC [73, 74] while reversal learning relies upon the orbitofrontal cortex (OFC) [75–77]. In addition, dynamic interactions between the OFC and hippocampus for reversal learning have been noted, with local field potentials of the OFC and hippocampus exhibiting coherence in theta rhythm in a performance-dependent manner [78, 79] and contralateral lesions of the OFC and ventral hippocampus in mice subtly, though significantly, impairing spatial reversal learning [78]. Systemic D-serine treatment has shown beneficial effects on spatial reversal learning [69, 80–83]. However, for this study, upregulation of SR in the mPFC did not affect reversal. Given that the lentiviral injection targeted the mPFC rather than the OFC, the lack of effects on reversal learning observed in this study aligns with the literature regarding the critical role of the OFC and hippocampus, rather than the mPFC, to this cognitive capacity.

By targeting the mPFC with LV-SR, enhancement in set-shifting performance would have been expected. However, no effects of manipulation were noted on this measure. Few studies have evaluated the impact of D-serine in set-shifting, but there is evidence of glutamatergic signaling impacting set-shifting and reversal learning performance. In adult rodents, disruption of medial prefrontal NMDAR or AMPA receptor function impaired set-shift performance, increasing perseverative errors, but had no effect on reversal learning [84–86]. More selective manipulations, targeting GluN2A and GluN2B specifically, have demonstrated their important role in extradimensional set-shifting and spatial reversal learning [80, 87–89]. In terms of aging, age-related reductions in GluN1 expression in the mPFC were correlated with impaired set-shifting performance in aged rats, while expression of AMPA receptor subunits that were found to be decreased with aging (GluR1, GluR2) did not relate to set-shifting performance [90]. Therefore, enhanced NMDAR-mediated function via purported increases in D-serine would be expected to improve set-shifting. Despite increased NMDAR-mediated transmission in the mPFC in this study, corresponding changes in set-shifting did not emerge. This may be due to lower levels of D-serine attained in this study (through the elevation of SR expression) than was observed or used in other studies. The exact levels of D-serine in the mPFC were not measured and is a limitation of this study.

LV-SR infusion in the mPFC accelerated learning in the visual discrimination condition, as noted by the reduced trials to criterion. In agreement with these findings, NMDAR hypofunction impairs visual discrimination, as observed with NMDAR antagonists [91–93], deletion of the GluN1 subunit on dopaminergic neurons [94], and GluN2A knockouts [95, 96]. This is the first study to demonstrate that enhanced basal NMDAR synaptic function, via viral vector-mediated upregulation of SR expression in mPFC of middle-aged rats, was associated with improved visual discrimination learning. The results from this study support the beneficial effects of the D-serine pathway involvement in NMDAR-mediated transmission and cognitive function, expanding the literature to emphasize its role in not only the hippocampus but also the PFC. Thus, targeting this pathway could pose a potential route in reversing age-related cognitive decline and should be considered for future research.

## MATERIALS AND METHODS

### Subjects

Middle-age male Fischer-344 rats (~12-month) were obtained from the National Institute on Aging (Bethesda, MD, USA) and housed at the University of Florida in a temperature- and humidity-controlled vivarium on a 12:12 light/dark cycle (lights on: 7am). Rats remained pair-housed with full access to food and water and habituated to the facilities for a week prior to handling. Once acclimated and handled, rats underwent surgery for injection of either green fluorescent protein (GFP) or SR lentiviral vector with a Ca^2+^/calmodulin-dependent protein kinase II (CaMKII) promoter into the mPFC. After recovering for five weeks, rats were food restricted to 85% of their original weight, trained and tested on the attentional set-shift task (AST), following which they were perfused and brains were collected and examined for verification of viral expression. All procedures involving animals were approved by the Institutional Animal Care and Use Committee at the University of Florida and were in agreement with guidelines recognized by the U.S. Public Health Service Policy on Humane Care and Use of Laboratory Animals. A schematic of experiment timeline is provided in Figure 1A.

### Lentiviral vector and mPFC injections

Lentiviral particles encoding SR (LV-SR) and green fluorescent protein (LV-GFP) were obtained from BioSource SAS-Genetic Engineering Technologies (GEG Tech, Paris, France). SR and GFP cDNAs were cloned into vectors containing a neuron specific promoter, CaMKII. Plasmids were packaged into vesicular stomatitis virus (VSV) glycoprotein envelope before stereotaxic injections into the mPFC of male middle-aged rats. Rats were anesthetized with isoflurane in the induction chamber, and stereotaxic techniques (KOPE Stereotaxic Alignment System) were employed for virus injection. Lentiviral vector encoding SR (LV-SR, ∼1.0 × 10^9^ transducing units/mL) or GFP (LV-GFP) was bilaterally injected into the mPFC (anterior/posterior +3.0 mm and medial/lateral ± 0.5 to 0.8 mm of bregma, dorsal/ventral 2.2 to 2.6 mm) using a glass pipette. Vector was injected bilaterally into mPFC and each injection consisted of ~2 μl of SR or GFP vector. One cohort of rats was utilized for the behavior and immunohistochemical analyses, while a separate cohort of rats was used for the electrophysiology.

### Attentional set shift task

#### Apparatus

The attentional set-shift task (AST) was conducted in rat operant boxes (Coulbourn Instruments, Whitehall, PA, USA), containing a food magazine port between two levers, which were located below two lights on the front panel. A house light was positioned on the rear wall. Operant boxes were individually stored within a sound-attenuating chamber with a fan for aeration and noise dampening. The input and output from each box were transmitted to Graphic State 4 software (Coulbourn Instruments) on an Optiplex 9020 computer.

#### Behavioral paradigm

Once rats fully recovered from surgery (~five weeks), they were food restricted to 85% of their starting body weight. Rats were exposed to the food reward pellets in their home cage during this period to reduce neophobia. The task was modified from Floresco et al., 2008 [74, 97, 98]. For two days, rats were habituated to the operant chamber for 15 min, with the back house light on and 10 food pellets in the magazine. Rats were trained to lever press, whereby they had 30 min to hit the extended levers on an FR-1 schedule for a food pellet reward. Once they pressed each lever 50 times, rats transitioned to sessions in which the levers were extended for 10 s for each trial. If no response was made, a “time out” period of 10 s occurred where the house light extinguished, and an omission was recorded. During the lever presentation, one of the two front panel lights was pseudo-randomly illuminated to expose the rats to this stimulus. Rats needed 90% performance before being evaluated for a side bias. During the side bias assessment, rats performed seven trials and had to alternate between each lever. Whichever lever they displayed a persistent preference for was not selected as their “Set shift” lever (see below). Rats progressed to a visual discrimination (VD) paradigm, in which the location of the light cue predicted the rewarded lever (the lever below the light). For criterion of VD and later stages (the extra-dimensional shift and reversal), rats needed to correctly perform 8 consecutive trials and greater than 30 trials total to progress to the next stage. Each session consisted of 120 trials maximum, and rats continued with the same phase until they reached criterion. The time out period remained at 10s and the inter-trial interval was 9–12s. After VD, rats made an extradimensional shift (set-shift) from the light cue as the predictor to an egocentric response, whereby the location of the lever itself (right or left of the rat) predicted the reward. Once rats attained criterion on the set-shift paradigm, the rewarding response was reversed (i.e., a reversal) to the opposite lever location.

#### Behavioral analysis

The number of trials to criterion served as a measure of the rats’ capacity to learn a new strategy, as well as make an extra-dimensional set shift and reversal. Total omissions, calculated as (trials omitted/total trials) × 100, were also measured. Performance errors were recorded and, during the set-shift paradigm, were characterized as perseverative, regressive, or never reinforced. Perseverative errors were defined as incorrect lever selection (i.e., below a light cue and thus perseverating to the VD strategy) when more than 70% of these errors were made. When fewer than 70% of these errors were made, the incorrect selections were defined as regressive errors, indicative of a gradual shift to the new response strategy. During VD and reversal stages, only total errors were calculated.

### Immunohistochemistry

#### Tissue collection

Rats were anesthetized with isoflurane and underwent transcardial perfusion with 200 mL of ice cold 1X phosphate buffered saline (PBS) and 4% paraformaldehyde (PFA). Brains were extracted, placed in 4% PFA for overnight incubation at 4°C, and transferred to 30% sucrose. After sinking in the solution, brains were embedded in OCT and maintained at –80°C until slicing. Brains were sliced on a cryostat (Microm, Waltham, MA, USA), as 40 μm-thick coronal sections, and the prefrontal cortical region was collected and maintained in cryoprotectant solution (30% ethylene glycol, 15% glucose, 0.04% sodium azide in 0.05M PBS).

#### Immunofluorescent procedure

Six sections of the PFC (A/P range: +4 to +2) were processed for co-localization of CaMKII, which was the promoter of both lentiviruses, and GFP, which served as an indicator of the viral vector infusion site for LV-GFP and LV-SR groups. As previously conducted [99] coronal sections were rinsed thrice for 5 min in 1X tris-buffered saline (TBS), incubated in antigen retrieval solution (Vector, Burlingame, CA, USA) for 10 min at 95°C, rinsed again, and blocked in 10% donkey serum for 1 hr. Sections were then incubated at 4°C for 48 hrs for mouse anti-CaMKII (1:1000, Invitrogen, Carlsbad, CA, USA) and 24 hrs for rabbit anti-GFP (1:500; Invitrogen) in 1% donkey serum. Slices were then rinsed in TBS and 1% Triton X100 and incubated in 1% donkey serum with AlexaFluor-488 donkey anti-mouse (1:500; Invitrogen) and donkey anti-rabbit-594 (1:500; Jackson, Westgrove, PA, USA). Slices were rinsed 3 × 5 min in TBS, mounted onto slides and cover slipped with DAPI (Vector).

An additional set of prefrontal slices underwent free-floating immunohistochemistry for SR expression, to confirm the efficacy of the LV-SR in increasing SR levels in the PFC. Slices were rinsed thrice for 5 min in 1X PBS, heated at 95°C for 10 min in antigen retrieval solution (Vector), rinsed, and blocked for 2 hrs in 10% donkey serum and 0.3% Triton in 1X PBS. Sections were incubated overnight at 4°C in 1% bovine serum albumin (BSA) in PBS with mouse anti-serine racemase (1:100, Santa Cruz, Dallas, TX, USA). Free-floating sections were rinsed thrice and incubated for 2 hrs in secondary antibody (donkey anti-mouse-488; 1:500; Invitrogen) in 1% BSA. Sections were rinsed, mounted onto slides and cover slipped with mounting media containing DAPI (Vector).

#### Image analysis

Slices fluorescently tagged for co-localization of GFP/CaMKII were imaged at 200× magnification, while SR-tagged slices were imaged at 400×, on a Leica DM2500 microscope (Wetzlar, Germany), equipped with a Retiga 4000R camera (QImaging, Surrey, BC, Canada) and QCapture Pro7 software (QImaging). Images were then enhanced for improved visualization using Adobe Photoshop (San Jose, CA, USA) prior to co-localization analysis, which was calculated as [(CaMKII+ and GFP+)/all GFP+] × 100. Approximately 100 GFP-positive cells at the infusion site were included for each animal. To evaluate SR expression, images were converted from pixels to micrometer (56pixels/μm) and enhanced to reduce background noise, and the total area with positive fluorescent tagging in the mPFC was calculated using NIH *ImageJ*. Tissue from LV-GFP (*N* = 9) and LV-SR (*N* = 7) were then analyzed for statistical differences in SR expression.

### Electrophysiology

#### mPFC slice preparation

The protocol for preparation of mPFC slices for electrophysiological studies were modified from standardized lab protocols [20–23, 30, 99–105]. Animals were deeply anesthetized using isoflurane and decapitated with a guillotine (MyNeurolab, St Louis, MO, USA). The brain was rapidly removed and transferred into a beaker containing ice-cold artificial cerebrospinal fluid (aCSF). The PFC was blocked and coronal slices (~400 μm) were cut using a tissue chopper (Mickle Laboratory Engineering Co, Surrey, UK). The freshly cut slices were incubated in a holding chamber (at room temperature) with aCSF containing (in mM): 124 NaCl, 2 KCl, 1.25 KH_2_PO_4_, 2 MgSO_4_, 2 CaCl_2_, 26 NaHCO_3_, and 10 D-glucose. Slices were transferred to a standard interface recording chamber (Warner Instrument, Hamden, CT, USA) at least thirty minutes before recording. The chamber was continuously perfused with oxygenated aCSF (95%-O_2_ and 5%-CO_2_) at the rate of 2 mL/min and the temperature was maintained at approximately 37°C using the TC-324B temperature controller (Warner Instrument, Hamden, CT, USA). The pH of the aCSF was maintained at 7.4.

#### Extracellular field potential recordings

Extracellular field excitatory postsynaptic potentials (fEPSPs) were recorded using a glass micropipette electrode filled with aCSF. The glass micropipettes were pulled from thin-walled borosilicate capillary glass using a Flaming Brown horizontal micropipette puller (Sutter Instruments, San Rafael, CA, USA), and the electrode resistances ranged from 4–6 MΩ. The recording pipette was localized to layer 2/3 of the mPFC (Figure 4). A concentric bipolar stimulating electrode (FHC, Bowdoinham, ME, USA) was localized to layer 4/5. Diphasic stimulus pulses (100 μsec, SD9 Stimulator, Grass Instrument Co., West Warwick, RI, USA) were delivered to layer 4/5 of the mPFC (0.033 Hz) to evoke fEPSPs. The signals were sampled at a frequency of 20-kHz, amplified and filtered between 1 Hz and 1 kHz using Axoclamp-2A (Molecular Devices, Sunnyvale, CA, USA) and a differential AC amplifier (A-M Systems, Sequim, WA, USA). Field potential data were stored on a computer hard drive (Dell Inc., TX, USA) for off-line analysis. A separate output from the differential AC amplifier was fed into an oscilloscope (Tektronix 2214, Tektronix Inc., Beaverton, OR, USA) for real time visualization of the signals. In order to measure the amplitude of the fEPSP, two cursors were placed to encompass the entire waveform. A Sciworks computer algorithm (Datawave Technologies, Berthoud, CO, USA) was used to compute the maximum amplitude (mV) of the fEPSP at the peak of the waveform, as well as the slope of the descending response. In order to measure the slope of the fEPSP, two cursors were placed around the initial descending phase of the waveform and the maximum slope (mV/ms) of the fEPSP was determined by a computer algorithm that found the maximum change across all sets of 20 consecutively recorded points between the two cursors. Input-output curves for the slope of the total fEPSP were constructed for increasing stimulation intensities.

#### Isolation of NMDAR-mediated synaptic response

Following collection of the total synaptic response, the NMDAR-mediated field excitatory postsynaptic potentials (NMDAR-fEPSPs) were isolated and recorded as described previously [20, 30, 99, 100, 104, 105]. In order to obtain the NMDAR-fEPSP, mPFC slices were incubated in aCSF containing a low concentration of extracellular Mg^2+^ (0.5 mM), 6,7-dinitroquinoxaline-2,3-dione (DNQX, 30 μM), and picrotoxin (10 μM). Input-output curves for the NMDAR-fEPSPs were constructed for increasing stimulation intensities. In some cases, pharmacological isolation of the NMDAR-fEPSPs was confirmed by the application of the NMDAR antagonist AP-5 (100 μM).

Our previous results demonstrate that redox state modulates NMDAR-mediated synaptic function in mPFC [20], so we investigated whether the increased expression of SR interacted with redox state to alter the NMDAR synaptic response. To examine the effects of the reducing agent, dithiothreitol (DTT), the baseline response was set at ~50% of the maximum, and responses were collected for at least 10 min before and 60–70 min after drug application. The DTT dose (0.5 mM) was selected due to previous studies that showed that this dose was within a range that can increase NMDAR responses in aged animals and in young animals under oxidizing conditions, yet is below a dose that impairs enzyme activity [21, 30, 106, 107].

### Statistical analysis

Examining the impact of LV-SR infusion into the mPFC, a one-way analysis of variance (ANOVA) was applied to the immunohistochemical and behavioral data, with infusion type as the between-subjects factor. Under conditions where homoscedasticity or normality were violated, the Brown-Forsythe or Mann-Whitney U statistic was applied, respectively. For electrophysiological recordings, repeated-measures ANOVAs, with stimulation as the repeated measure, were conducted. Significant differences were localized using Fischer’s PLSD *post hoc* comparisons (*p* < 0.05), and data were interpreted as significant if *p* ≤ 0.05.
